# An Open Discussion Invited

**Published:** 1918-12-07

**Authors:** 


### A STATE MEDICAL SERVICE.

### An Open Discussion Invited.

THE publication in THE HOSPITAL of "A Vision of State Medical Service," by Colonel G. T. K. Maurice, C.M.G., has attracted some attention. Indeed it is reported that a large number of members of the profession on service at the Front have shown much interest in the "Vision," which has been a constant theme of discussion amongst them. In accordance with the wishes expressed "A Vision of State Medical Service" has now been published separately as a pamphlet, which may be obtained through the usual channels, or direct from the publishers, The Scientific Press, Ltd., 28 Southampton Street, Strand, London, W.C. 2 (1s.).

The interest is evidently spreading if we may judge from the communications we are receiving. An exhaustive discussion covering all views and sides of this question will be welcomed by many practitioners of all ranks. To-day we have the pleasure to publish two communications, the first of which embodies some views of a distinguished physician, whilst the second contains a summary of an article by Dr. W. A. Brend, published in the *Practitioner* of November.

